# Anatomical Variants in Pancreatic Irrigation and Their Clinical Considerations for the Pancreatic Approach and Surrounding Structures: A Systematic Review with Meta-Analysis

**DOI:** 10.3390/medicina61040666

**Published:** 2025-04-04

**Authors:** Juan José Valenzuela-Fuenzalida, Camila Ignacia Núñez-Castro, Valeria Belén Morán-Durán, Pablo Nova-Baeza, Mathias Orellana-Donoso, Alejandra Suazo-Santibáñez, Alvaro Becerra-Farfan, Gustavo Oyanedel-Amaro, Alejandro Bruna-Mejias, Guinevere Granite, Daniel Casanova-Martinez, Juan Sanchis-Gimeno

**Affiliations:** 1 Departamento de Morfología, Facultad de Medicina, Universidad Andrés Bello, Santiago 8370146, Chile; juan.kine.2015@gmail.com (J.J.V.-F.); cam.nunezcas@gmail.com (C.I.N.-C.); vale260902@gmail.com (V.B.M.-D.); pablo.nova@usach.cl (P.N.-B.); alejandro.bruna@upla.cl (A.B.-M.); 2 Escuela de Medicina, Universidad Finis Terrae, Santiago 7501015, Chile; mathor94@gmail.com; 3 Department of Morphological Sciences, Faculty of Medicine and Science, Universidad San Sebastián, Santiago 7510157, Chile; 4 Faculty of Health and Social Sciences, University of the Americas, Santiago 8370040, Chile; alej.suazo@gmail.com; 5 Departamento de Ciencias Química y Biológicas, Facultad de Ciencias de la Salud, Universidad Bernardo O’Higgins, Santiago 8370993, Chile; alvaro.becerra@ubo.cl; 6 Facultad de Ciencias de la Salud, Universidad Autónoma de Chile, Santiago 8910060, Chile; g.oyanedelamaro@gmail.com; 7 Departamento de Ciencias y Geografía, Facultad de Ciencias Naturales y Exactas, Universidad de Playa Ancha, Valparaíso 2360072, Chile; 8 Department of Surgery, F. Edward Hebert School of Medicine, Uniformed Services University of the Health Sciences, Bethesda, ML 20814, USA; guinevere.granite@usuhs.edu; 9 Facultad de Medicina, Universidad de Valparaíso, Campus San Felipe, Valparaíso 2170000, Chile; daniel.casanova@uv.cl; 10 Laboratorio de Neuroanatomía Microquirúrgica (LaNeMic), Facultad de Medicina, Universidad de Buenos Aires, Buenos Aires C1053, Argentina; 11 GIAVAL Research Group, Department of Anatomy and Human Embryology, Faculty of Medicine, University of Valencia, 46001 Valencia, Spain

**Keywords:** pancreas vasculature, variations of irrigation of pancreas, aberrant irrigation of pancreas, clinical anatomy, pancreatic anatomy, pancreatic surgery, anatomical variations

## Abstract

*Background and Objectives*: The pancreas receives blood through a complex network of multiple branches, primarily originating from the celiac trunk (CeT) and the superior mesenteric artery (SMA). This blood supply is structured into three main arterial groups, each serving different regions of the pancreas to effectively support its endocrine and exocrine functions. *Materials and Methods*: The databases Medline, Scopus, Web of Science, Google Scholar, Cumulative Index to Nursing and Allied Health Literature (CINAHL) and Latin American and the Caribbean Literature in Health Sciences (LILACS) were searched until January 2025. Methodological quality was evaluated using an assurance tool for anatomical studies (AQUA). Pooled prevalence was estimated using a random effects model. *Results*: A total of sixteen studies met the established selection criteria in this study for meta-analysis. Pancreatic irrigation variants presented a prevalence of 11.2% (CI: 7–14%) and a heterogeneity of 88.2%. The other studies were analyzed by subgroups, showing statistically significant differences in the following subgroups: (1) sample type—a larger sample of images analyzed in the included studies (*p* = 0.312), which did not show statistically significant differences; (2) geographical region (*p* = 0.041), which showed a greater presence in the Asian population studied, and this was statistically significant; and (3) sex (male or female) (*p* = 0.12), where there were no statistically significant differences. *Conclusions*: The discovery of variations in pancreatic irrigation is common due to the numerous blood vessels involved in supplying this vital organ. Understanding different vascular patterns (such as those from the splenic and mesenteric arteries) is crucial for surgical interventions on the pancreas. For transplant patients, a thorough vascular analysis of both the donor and recipient is essential. Variations can impact blood flow and compatibility, potentially leading to transplant rejection if not addressed. To enhance outcomes, it is recommended to develop more accurate imaging tools for pre-surgical analysis, necessitating ongoing research in this area.

## 1. Introduction

The arterial supply of the pancreas is a complex, multibranched system primarily derived from the celiac trunk (CeT) and the superior mesenteric artery (SMA). It is organized into three main arterial constitutions, which supply different portions of the organ, ensuring adequate blood flow for its endocrine and exocrine functions [1]. However, anatomical variations in the pancreatic arterial supply are common, with origins also observed from the splenic artery, common hepatic artery, and SMA, among others. Understanding these variations is crucial for performing safe pancreatic resections and minimizing complications during surgical procedures [1].

The anatomical variations in the pancreatic arterial supply significantly influence surgical resections, particularly in procedures like the Whipple operation. Understanding these variations is crucial for minimizing complications and optimizing surgical outcomes [2].

The right aspect of the arterial system consists of the pancreaticoduodenal arcade, which has key structures that irrigate the head of the pancreas. One of its most prominent branches is the anterior superior pancreaticoduodenal artery (ASPDA), which descends vertically from the gastroduodenal artery (GDA) to the lower edge of the pancreas. In addition, the anterior limb of the inferior pancreaticoduodenal artery (IPDA), originating from the SMA, follows an anterior, downward, and rightward path, joining with the pancreatic arterial arch and contributing to the blood supply of this region [3,4].

The middle region of the pancreas is primarily supplied by the dorsal pancreatic artery (DPA), one of the prominent branches of the splenic artery (SA). This artery, which can reach up to 1 cm in diameter, whilst it is generally between 1 and 3 mm, follows a caudal path until it divides into a right and a left branch at an inverted “T”-shaped intersection. The right bundle branch connects to the posterior pancreaticoduodenal arcade in 40–50% of cases, while the left bundle branch, referred to as the transverse pancreatic artery (TPA), is a constant terminal branch that contributes to the central region of the pancreas [3,4]. The left aspect of the arterial system includes the arteries that descend from the splenic artery (SA) into the body and tail of the pancreas. Among these is the greater pancreatic artery (GPA), which descends vertically down the posterior aspect of the pancreas and can anastomose with the TPA in some cases. Additionally, the left branch of the DPA extends to the tail of the pancreas, providing extensive irrigation to this distal region of the organ [3,4].

The embryological development of pancreatic drenching is closely related to the formation of the pancreas from the ventral and dorsal buds of the foregut. These buds, formed between the fifth and eighth week of gestation, are derived from endodermal cells that proliferate in the caudal region of the foregut. The dorsal yolk appears first on the posterior aspect of the duodenum, while the smaller ventral bud arises near the common bile duct [4].

In a pioneering study by Woodburne et al. (1951) [5], the incidences and variations in pancreatic arteries were examined, providing a comprehensive view of their origin and distribution in the vascular system. It was observed that the ASPDA has an invariable origin from the GDA, with an incidence of 100% in the research series, confirming its constancy without reported variations. This finding has been validated by almost all previous research, reinforcing its predictability in surgical procedures [4]. On the other hand, the superior posterior pancreaticoduodenal artery (SPDA), although it presents a slight variability in comparison, shows a high incidence (99.3%) in the Woodburne et al. (1951) [5] studies. This percentage is similar to studies by other researchers, such as Petrh (2013) (100%), Edwards (1997) (97%), and Pierson (2001) (96%). Most cases (92.6%) indicate that this artery originates from the GDA, even though in a small percentage (3%), it has been observed that it comes from the right hepatic branch accessory to the SMA. Its anatomical position is usually in front of the common bile duct, except in less common cases where its origin is behind the pancreatic head [6]. In a study by Rousek et al. (2022) [7] on the DPA, 31 investigations were analyzed to evaluate the presence and origin of the artery, identifying 95.8% of 2322 total cases, with a prevalence of 95.1% in anatomical samples (1233 cases) and 96.4% in radiological explorations (1089 cases), with no significant differences between the two methods. The most common origins of PDA were the SA (37.6%), the SMA (23.9%), the common hepatic arteries (18.3%), and the CeT (11.9%), with other less frequent origins such as aberrant hepatic arteries, GDA, and the middle colic artery (MCA), which accounted for 2.77% combined. The diameter of the artery ranged from 1 mm to 1 cm, with 1–3 mm being the most common average. Additionally, the presence of multiple dorsal pancreatic arteries was documented in anatomical and radiological studies, with double arteries reported in up to 18% and triple arteries in 5% of cases [7]. The classification by Yamane et al. (2023) identifies ten anatomical types of the DPA, highlighting the diversity in their origin and their relationship with the accessory middle colic artery (AMCA). The main origins of DPA include the SA, the SMA, and the CeT [1]. Alternatively, the classification by Okahara et al. (2010) is based on the analysis of cross-sectional imaging inquiries, identifying anatomical variants in the main pancreatic arteries. This approach describes specific patterns, such as pre-pancreatic and retro-pancreatic anastomosis arches [8]. Among the postoperative complications associated with the vascular supply, the literature reports that one of the most frequent vascular conditions is postoperative pancreatic fistula (POPF), with an incidence that ranges between approximately 3%. Therefore, an adequate approach could depend on surgeries and whether the medical team has vascular knowledge, both of normal anatomy and of the variants in pancreatic irrigation, and knowing these variants will be key for diagnostic surgeries with imaging support of the irrigation structures of the pancreatic area and surrounding areas [1,8]. Furthermore, anatomical variations influence complications such as ischemia that affect the normal course of the bloodstream, which, if associated with distribution vessels, and if these are hypoplastic in relation to normal anatomy, could cause ischemia processes in the irrigation area. They could also cause hemorrhage, since their layers associated with the endothelium could be of smaller caliber and more susceptible to rupture. Finally, if anastomosis approaches must be made in surgery, these could fail if the most common or rare variants during pancreatic procedures are not known [3,8,9].

The objective of this review and meta-analysis was to determine the prevalence and anatomical characteristics of pancreatic irrigation with variants and how they are associated with clinical considerations in the abdominal organs.

## 2. Methods

### 2.1. Protocol and Registration

This systematic review and meta-analysis adhered to the guidelines outlined in the Preferred Reporting Items for Systematic Reviews and Meta-Analyses (PRISMA) statement [10]. The registration number in the International Prospective Register of Systematic Reviews (PROSPERO) is CRD42024520734.

### 2.2. Eligibility Criteria

The studies included in this review were selected based on specific criteria to ensure methodological rigor and adherence to PRISMA guidelines. The population criteria encompassed samples from donor dissections and live images of VA variations. The selected studies examined the prevalence of pancreatic irrigation variants and their correlation with abdominal organ pathologies, with a particular focus on pancreatic irrigation abnormalities. Anatomical variants were classified and described according to normal anatomy, existing classifications, and literature-based descriptions.

To maintain the quality and relevance of the review, only research articles and case reports involving human samples, published in English in peer-reviewed journals, and indexed in the reviewed databases were included. Letters to the editor and studies involving animal samples were excluded to minimize bias and ensure clinical applicability. This rigorous selection process strengthens the validity of the systematic review and meta-analysis, ensuring that the findings are based on high-quality and relevant evidence.

### 2.3. Electronic Search

The systematic literature search was conducted using the following databases: MEDLINE (via PubMed), Web of Science, Google Scholar, the Cumulative Index to Nursing and Allied Health Literature (CINAHL), Scopus, and Latin American and the Caribbean Literature in Health Sciences (LILACS) until January 2025. The search strategy incorporated a combination of the following terms: “pancreas arteries” (no mesh), “variations irrigation of pancreas” (no mesh), “aberrant irrigation of pancreas” (no mesh), “clinical anatomy” (no mesh), “surgery pancreas” (no mesh), and “variations anatomical” (no mesh), using the Boolean operators AND, OR, and NOT. Detailed search strategies for each database are available in the Supplementary Materials.

The selection of these databases was based on their international recognition and extensive coverage of the health sciences literature, ensuring a comprehensive collection of relevant studies. MEDLINE and Web of Science provide access to high-impact biomedical literature, CINAHL covers nursing and allied health research, Scopus offers broad multidisciplinary coverage, LILACS includes valuable Latin American studies, and Google Scholar supplements the search with gray literature. This strategic selection maximizes the inclusion of pertinent research and minimizes publication bias.

### 2.4. Study Selection

Two of the authors (Moran V and Valenzuela J) independently screened the titles and abstracts of references obtained from the searches. The full text was obtained for references that either author considered potentially relevant. In cases where a consensus was not reached, a third reviewer (Orellana M) was involved. The inter-evaluator validity was assessed using the Kappa index, yielding a value of 0.78.

### 2.5. Data Collection Process

Two authors (MO and JS) independently extracted data on the outcomes of each study. The information obtained from the original reports included: (i) author and year of publication, (ii) region, (iii) N and sample, (iv) age and sex, (v) prevalence, (vi) clinical history, (vii) symptoms, (viii) artery with variants, (ix) description of the variants, and (x) clinical implications.

### 2.6. Assessment of the Methodological Quality of the Included Studies

For the assessment of bias, we have only performed a quality assessment using the methodological quality assurance tool for anatomical studies (AQUA), as proposed by the International Evidence-Based Anatomy Working Group (IEBA) [11]. Both data extraction and quality assessment were performed independently by two reviewers (JJV and CR). In cases where consensus was not reached, a third reviewer (JSG) was involved. The agreement rate between the reviewers was calculated using Kappa statistics (0.71).

### 2.7. Statistical Methods

The data extracted from the meta-analysis were processed using the R statistical software (https://www.r-project.org/, accessed on 1 October 2024) to calculate the prevalence of pancreatic irrigation variants. The combination of the summarized data was carried out using the DerSimonian–Laird model together with the Freeman–Tukey double arcsine transformation. Given the high level of heterogeneity in the prevalence of pancreatic irrigation variants, a random effects model was used.

To assess heterogeneity between the included studies, the chi^2^ test and the I^2^ statistic were applied. Following the recommendations of the Cochrane collaboration, a *p* value of 0.10 was considered significant in the chi^2^ test. The interpretation of the I^2^ statistic with a 95% confidence interval (CI) was as follows: 0–40% indicates non-significant heterogeneity, 30–60% suggests moderate heterogeneity, 50–90% reflects substantial heterogeneity, and 75–100% indicates considerable heterogeneity.

To examine the possible presence of the small study effect (a phenomenon in which smaller studies may show different results than larger ones), a DOI graph was generated accompanied by the LFK index [12,13].

### 2.8. Subgroup Analysis

To avoid biases that may lead to underestimating or overestimating the differences in results between subgroups, we applied the same statistical analysis previously mentioned to each of them. In addition, in the statistical analysis we incorporated the prevalence corresponding to each subgroup and clinical considerations in the qualitative analyses. Finally, we classified the subgroups into three categories—image sample, living patients and donor samples—performing an individual analysis for each one.

## 3. Results

### 3.1. Included Articles

**Figure 1:**
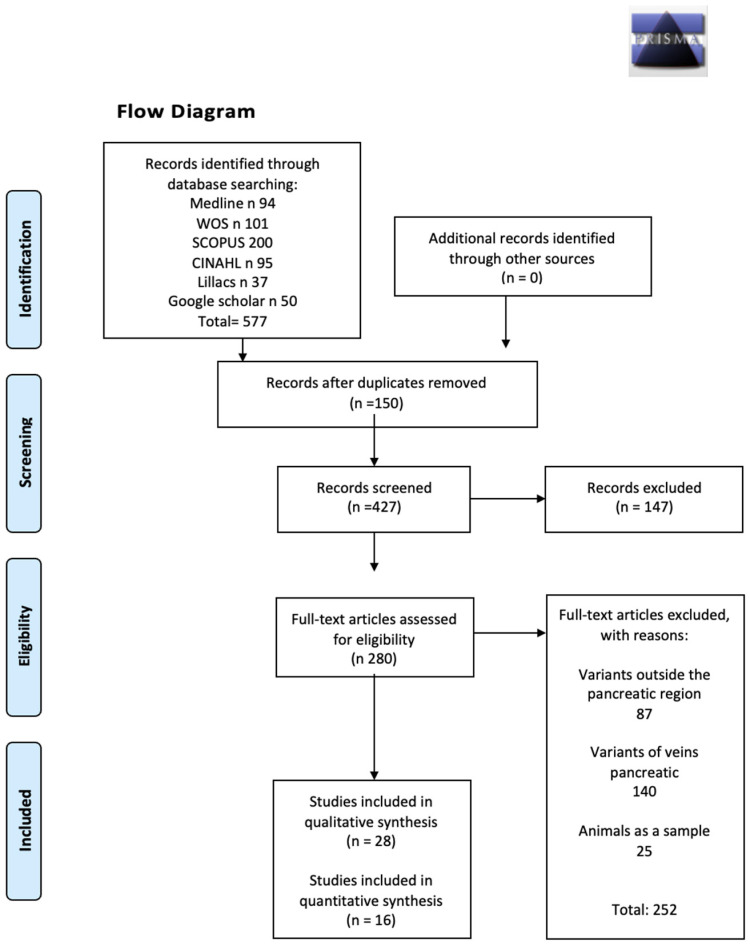
PRISMA flow diagram illustrating the identification, screening, eligibility, and inclusion process for studies in a systematic review.

A total of 577 articles from different databases met the criteria and search terms established by the research team. The filter was applied to the titles and/or abstracts of the articles in the databases consulted, and duplicates were removed. Subsequently, 280 full-text articles were assessed for eligibility for inclusion in this meta-analysis and systematic review. Fifty-five studies were excluded due to discrepancies between their primary and secondary outcomes compared with those of this review, or because they did not meet the established criteria for good data extraction. Therefore, twenty-eight studies [1,6,8,14,15,16,17,18,19,20,21,22,23,24,25,26,27,28,29,30,31,32,33,34,35,36,37,38] were considered eligible and included in this study (*n* = 1870). For the meta-analysis, the n was sixteen studies [1,6,8,18,24,25,26,27,28,29,30,32,33,34,35,36], with an n of 1551 (patients, images and donors) (Figure 1).

### 3.2. Characteristics of the Studies and Population

Of the 28 studies included in this review (Table 1), 13 articles are from Asia [1,8,14,24,25,26,27,28,29,32,34,36,37], eight are from Europe [15,19,20,21,30,31,33,38], seven are from America [6,16,17,18,22,23,35]. The mean age could not be estimated, and the types of studies included were observational and case series. Finally, the sex distribution was as follows: 477 men and 327 women; the sex of the remaining n was not declared in the studies, meaning the sex data provided do not cover the entire study population.

### 3.3. Variants Description

There are hormones that increase NO production, such as estrogen, progesterone, insulin, and growth hormones. They do so through both common mechanisms, which aid vascular growth and endothelial proliferation in large- and medium-caliber blood vessels. Conversely, some hormones, such as glucocorticoids, progesterone, and prolactin, decrease nitric oxide bioavailability. This is due to the variability in risk factors, such as cardiac vascular disorders, and even factors that could enhance vascular anatomical variants [41].

#### 3.3.1. Variability in the Inferior Pancreaticoduodenal and Dorsal Pancreatic Arteries

The IPDAs generally emerge from a common trunk of the SMA or from its superior jejunal branches, showing an incidence close to 99% for the anteroinferior artery of this system. Anatomically, these arteries are located behind the head of the pancreas or the uncinate process. In contrast, the DPA presents remarkable variability in terms of its origin. Previous researchers report rates ranging from 50% (Rio-Branco) to 90% in the current study, while others, such as Romodanowskaja, report 56%. This artery also presents diversity in its sources of origin, including the SA and the CeT, with 5% of cases interacting with the MCA [6,26].

#### 3.3.2. Inferior Pancreatic Artery and Classification of Variants According to Yamane et al. (2023) 

In Yamane et al. (2023)’s [1] study, DPA was investigated in a group of 101 patients, where a wide variety of origins and ramifications were identified. DPA showed a significant incidence of origin in different arteries, with the SA predominating (31%), followed by the CHA (17%) and the CeT (10%). Other origins included the SMA (27%), the replaced right hepatic artery (RRHA) (7%), the IPDA (5%), and some less common arteries (3%). This study also highlighted the complexity and diversity of the anatomical variations in DPA and its interaction with the AMCA, the latter being observed in 12% of cases. Furthermore, Buhler and Riolan vascular arches were recorded in two patients each, which showed the importance of anastomotic vessels in the path of DPA and AMCA.

**Figure 2:**
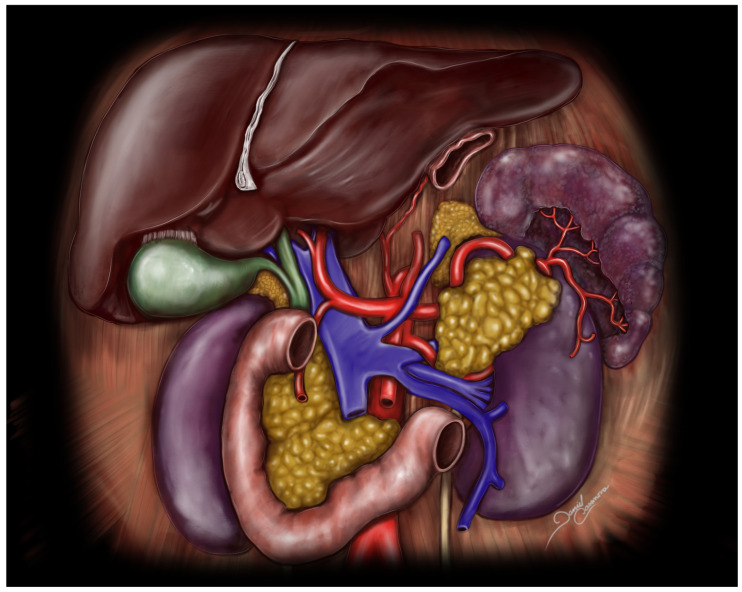
Anterior view. Arterial supply of the pancreas. The illustration depicts the main vascular structures supplying the pancreas, (Figure edited of Rousek et al., 2022 [9]).

Yamane et al. (2023) [1] identified ten anatomical types of the DPA and their relationship with the AMCA. In Type 1 (43%), DPA originates in the CeT or its branches, without AMCA. In Type 2 (16%), DPA also comes from CeT, but AMCA branches off from the SMA. Type 3 (3%) shows AMCA as a branch of DPA, while Type 4 (21%) has AMA-originated DPA without AMCA. In Type 5 (9%), the DPA arises from the SMA and the AMCA depends on it. In Types 6 and 7 (3% and 1%, respectively), DPA originates from the IPDA, in one with AMCA and in one without. Types 8 and 9 (1% each) feature the Riolan arch and Buhler’s arch, respectively, as collateral anatomical connections, while Type 10 (1%) also has the Buhler’s arch, but connects the CeT and SMA, offering additional collateral circulation routes (Figure 2).

#### 3.3.3. Okahara et al. (2010) Findings: Cross-Sectional Imaging and Pancreatic Variability

Okahara et al. (2010)’s [8] study analyzed cross-sectional arteriographic images to assess variations in major pancreatic arteries. He found that in 162 celiac arteriograms, a RRHA was present in fifteen cases and a replaced CHA in three cases. Moreover, Okahara et al. (2010) described the variation in the SPDA, with 14.2% of cases showing an anastomotic connection with the DPA (prepancreatic arch). The SPDA arose predominantly from the GDA, with 11.7% of cases presenting a retropancreatic arch. These anatomical patterns are relevant for surgical interventions, given that the anastomotic arches around the pancreas are key routes for maintaining irrigation [8].

#### 3.3.4. Pancreaticoduodenal Artery

**Figure 3:**
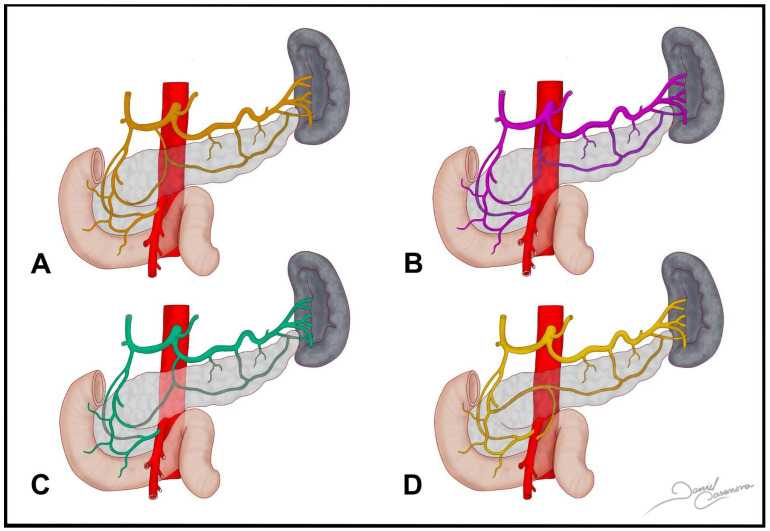
(**A**) Dorsal pancreatic artery from the common hepatic artery. (**B**) Dorsal pancreatic artery from the coeliac trunk. (**C**) Dorsal pancreatic artery from the splenic artery. (**D**) Dorsal pancreatic artery from the superior mesenteric artery.

The pancreaticoduodenal artery is an elementary vessel in the irrigation of the pancreas and duodenum, forming part of the complex arterial system that supplies these structures. It is divided into two main arteries: the SPDA, which is a branch of the GDA; and the IPDA, which originates from the SMA. The SPDA is subdivided into anterior and posterior branches, which run along the anterior and posterior aspects of the head of the pancreas and duodenum. These branches generate collaterals that contribute to the irrigation of the pancreas and the proximal segment of the duodenum, before anastomosing with the corresponding branches of the IPDA (Figure 3) [14,18].

### 3.4. Analysis of Prevalence and Subgroups

**Figure 4:**
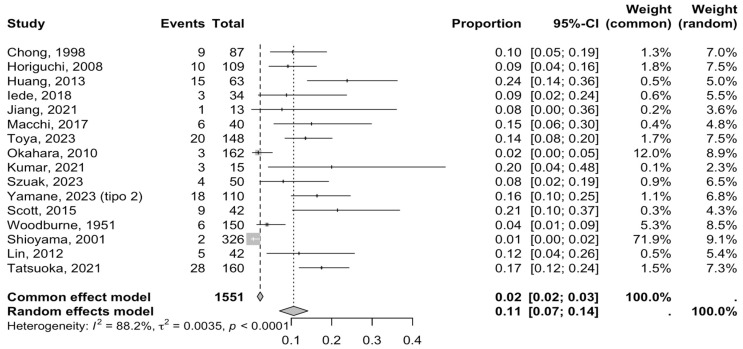
Forest plot of the prevalence of variants in pancreatic irrigation [1,6,8,18,24,25,26,27,28,29,30,32,33,34,35,36].

**Figure 5:**
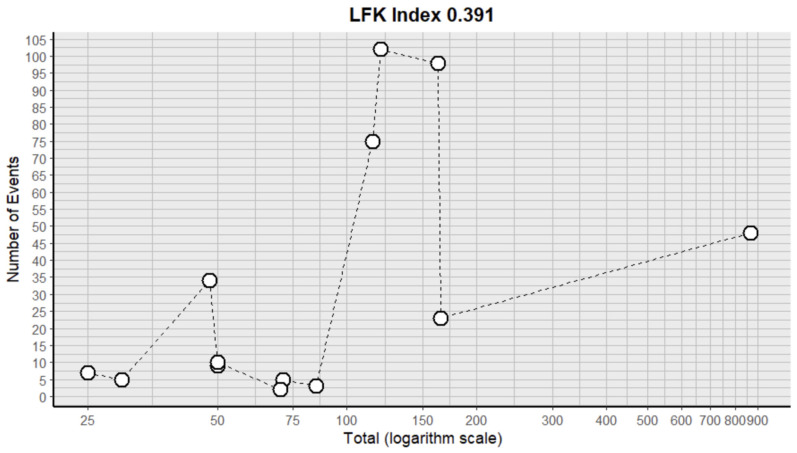
DOI plot with LFK index of prevalence for variants in pancreatic irrigation.

To calculate the prevalence of variants in pancreatic irrigation in the studies included in this review, four proportion forest plots were created. Sixteen studies [1,6,8,18,24,25,26,27,28,29,30,32,33,34,35,36] (Table 2) were included for the prevalence of irrigation pancreatic variants, presenting a rate of 11% (CI: 4% to 14%). The heterogeneity of the included samples was 88.12%, which is high, and the sample was quite heterogeneous between the groups analyzed with *p*-value *p* < 0.0001 (Figure 4 and Table 3). Although heterogeneity is high, in order to partially eliminate this limitation in the grouping of studies, we performed a subgroup analysis and we were able to understand that heterogeneity is more associated with the difference in n in the samples of the different studies included for the anatomical. However, the DOI graph with LFK index revealed a value of 0.391, indicating high skewness in the data published in the proportion meta-analysis, indicating a potential bias of our results (Figure 5).

Regarding the subgroup analysis, we have grouped studies with a prevalence of no more than 50%. The first subgroup consisted of donors and images. For the donor subgroup, five studies were included [6,27,28,30,33], with a prevalence of 14.0% (CI: 12.12–17.11%) and a heterogeneity of 79.11%. Among the imaging studies, 10 were included [1,8,18,24,25,26,29,30,32,34,35,36], with a prevalence of 8.84% (CI: 6.99–10.12%) and a heterogeneity of 94.85%. For this subgroup analysis, no statistically significant difference (*p* = 0.312) between image samples and donor samples was found. The second subgroup analysis was for the continents in which the included studies originated. From Asia, eleven studies were included [1,8,24,25,26,27,28,29,32,34,36], which presented a prevalence of 10.8% (CI: 8.87–11.99%) and a heterogeneity of 77.12%. From Europe, two studies were included [30,33], with a prevalence of 1.01% (CI: 0.77–2.12%) and a heterogeneity of 89.77%. From America, three studies were included [6,18,35], showing a prevalence of 2.42% (CI: 1.41–3.99%) and a heterogeneity of 93.12%. From Oceania and Africa, no studies were included. This subgroup showed statistically significant differences in favor of a greater presence of subjects with variations in pancreatic irrigation in the Asian continent. (*p* = 0.0041).

Regarding gender, first, for females, teen studies were included [1,8,26,27,29,30,33,35,36], with a prevalence of 8.31% (CI: 7.11% TO 9.56%) and a heterogeneity of 80.11%. For males, teen studies [1,8,25,26,27,29,30,34,35,36] were included with a prevalence of 4.42% (CI: 2.92–6.11%) and a heterogeneity of 78.12%. For this subgroup, there were no statistically significant differences (*p* = 0.12). Clinically, the existence of homology in the presence of male and female subjects with variants in pancreatic irrigation allows us to highlight that there is no association with sex, which suggests that practitioners should not worry about a distinguishing factor for pancreatic surgeries.

### 3.5. Risk of Bias of Included Articles

**Figure 6:**
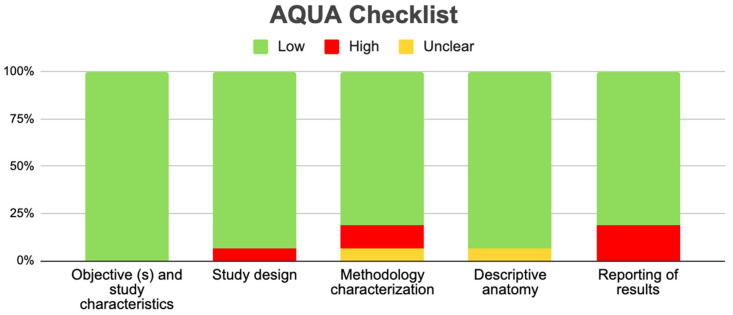
Bias risk assessment using the AQUA tool for studies included in the meta-analysis.

All studies included in the meta-analysis met the evaluation criteria using the AQUA checklist for anatomical studies, which assessed bias in five domains. The sixteen included studies presented a low risk of bias in domain 1 (objective and study characteristics). For domain 2 (study design), only one study presented a high risk of bias [6]. In domain 3 (methodology characterization), two studies presented a high risk of bias [25,33] and one study was unclear [35]. For domain 4 (descriptive anatomy), only one study was unclear [6]. Finally, for domain 5 (results reporting), three studies presented a high risk of bias [18,27,36] (Figure 6).

### 3.6. Clinical Considerations

The IPDA, which originates as a branch of the SMA, plays a critical role in the supply of the pancreas and duodenum. However, the presence of an aneurysm in this artery can trigger intestinal angina, manifesting itself with abdominal pain and weight loss, which may require surgical or endovascular intervention to prevent rupture [17,25]. In addition, there are significant anatomical variations, such as the presence of a RRHA, which instead of originating from the CHA, originates from the IPDA. This variant can complicate liver surgeries by modifying the usual blood supply and may be associated with multiple additional renal arteries, such as accessory renal arteries and additional renal arteries, which should be considered in liver and kidney procedures, including transplantation [19].

In the context of the diagnosis and treatment of pancreatic cancer (PC), the identification of secondary pancreatic arteries is essential, as they allow for a correct staging of pancreatic ductal adenocarcinomas. These vascular structures are key to assessing tumor extension and planning specific oncological interventions [18]. Conversely, the presence of an accessory right hepatic artery (ARHA) means a contraindication for simultaneous liver and pancreas transplants due to the risk of thrombosis and the difficulty in achieving adequate vascular reconstruction [25]. Another relevant artery is the CHA, which may be the origin of the SPDA. This artery is critical for blood supply to the pancreas and knowledge of it is vital in interventions such as the infusion of protease inhibitors for acute pancreatitis or in the administration of targeted chemotherapy for pancreatic carcinomas. Variations in the origin of the pancreaticoduodenal artery also pose a potential risk during surgical procedures, due to the alteration in blood flow distribution and the challenges involved in planning interventions with abnormal vascular anatomy [8].

Along with that, the inferior pancreatic artery (IPA) has significant anatomical variability, which must be considered during pancreatic surgical procedures to ensure adequate blood supply. Arterial arches related to this artery may be compromised during procedures if the specific anatomy is not taken into account, which could result in insufficient blood flow to the pancreas. Among these arteries are the ASPDA and the posterior superior pancreaticoduodenal (PSPDA) arteries, which are branches of the GDA and essential for the irrigation of the pancreas and duodenum. Its relevance is evident in cases of invasive pancreatic carcinoma, where ASPDA and PSPDA delimit the extent of the tumor, which can be observed by angiographic studies to evaluate tumor invasion [32].

The DPA is nevertheless one of the main irrigation vessels for the head and neck of the pancreas. Variations in their origin and trajectory can affect surgical technique and the preservation of blood flow, so it is essential to identify these characteristics during tumor resection and transplantation procedures. In addition, in interventions such as the Whipple duodenocephalopancreatectomy, identification of the right gastroepiploic artery (RGEA), which originates from DPA, is crucial for the success of the surgery [27].

PC is a highly aggressive neoplasm that is usually diagnosed in advanced stages due to a lack of symptoms in its early stages. Given its clinical behavior, surgical treatment remains the only potentially curative option, especially in cases where the tumor is resectable. The feasibility of resection, however, depends on the local extent of the disease and the potential invasion of nearby vascular structures, such as the SMA and portal vein. The criteria for determining resectability include the absence of significant vascular invasion and the absence of distant metastases, classifying tumors as resectable, borderline, resectable, and unresectable [42].

The surgical approach varies depending on the location of the tumor within the pancreas. Pancreatoduodenectomy—known as the Whipple procedure—is the most common intervention in tumors located in the head of the pancreas and involves the resection of several anatomical structures, including the pancreatic head, duodenum, and a portion of the stomach, among others. The proximity of the tumor to critical vascular structures makes presurgical evaluation of arterial irrigation critical to planning safe resection and reducing intraoperative complications, such as bleeding and vascular injury. Subsequent reconstruction using pancreatic, biliary, and digestive anastomoses represents another challenge, especially when there are anatomical variants in the irrigation of the pancreas [42].

In tumors located in the body or tail of the pancreas, distal pancreatectomy is chosen, which usually involves concomitant removal of the spleen. Although this surgery is less complex than pancreatoduodenectomy, the risk of exocrine and endocrine pancreatic insufficiency is still significant. Preservation of arterial blood supply in these cases is crucial, as anatomical variations can compromise blood flow to the remaining portions of the pancreas, increasing the risk of ischemia and postoperative pancreatic dysfunction [43].

Total pancreatectomy, even if it is infrequent, is indicated in diffuse tumors or in cases of chronic pancreatitis concomitant with cancer. This procedure carries a high risk of total pancreatic insufficiency, requiring continuous replacement of insulin and pancreatic enzymes. Complete loss of the pancreas requires accurate knowledge of arterial irrigation, since the preservation of critical vascular structures can influence the success of surgery and the patient’s hemodynamic stability during the postoperative period [42,43].

Postoperative complications of PC remain a clinical challenge. Among them, anastomosis leaks, bleeding and infections are the most common, and many of these complications are related to the proximity of the pancreas to key arterial vessels. In particular, anatomical variations in the pancreatic artery may predispose to iatrogenic lesions during resection, affecting surrounding vascular structures and compromising the perfusion of residual pancreatic tissue. This highlights the importance of adequate presurgical identification of vascular variants by advanced imaging studies, like angiography and contrast-enhanced computed tomography (CT) [41,42]. The postoperative prognosis depends on several factors, including the extent of the cancer, obtaining negative margins, and response to adjuvant treatment. Even with complete resection, tumor recurrence remains common, with five-year survival rates of approximately 20–25%. In this context, surgical planning based on detailed knowledge of pancreatic arterial irrigation plays a fundamental role in minimizing complications, improving postoperative outcomes, and optimizing long-term follow-up of these patients [42,43].

As in PC, duodenal cancer (DC) presents significant surgical and postoperative challenges. While less common than other gastrointestinal cancers, their surgical management depends on the location and extent of the tumor, as well as its relationship with key vascular structures. The proximity of the duodenum to the pancreas and to large vessels means that surgical planning requires a detailed evaluation of arterial blood supply in order to minimize intraoperative and postoperative complications. In cases where the tumor involves the head of the pancreas and the first portion of the duodenum, pancreaticoduodenectomy (Whipple procedure) is the intervention of choice. Due to the complexity of reconstruction, which includes enteric, hepatic-jejunal, and gastroenteric pancreatic anastomosis, the identification of anatomical variants in pancreatic arterial irrigation is critical to reduce the risk of vascular complications, such as pancreatic or biliary hemorrhages and fistulas [44]. When the tumor is located in more distal segments of the duodenum without affecting the pancreas, a partial duodenectomy may be chosen, preserving the adjacent structures. In these cases, duodenojejunostomy allows the continuity of gastrointestinal transit to be maintained. However, the feasibility of these procedures depends on the integrity of the arterial supply of the remaining segments, as variations in blood flow can compromise healing and increase the risk of postoperative ischemia [45].

The main goal of surgery in DC is to achieve negative resection margins, as this directly influences prognosis. Preoperative evaluation by CT, magnetic resonance imaging (MRI), and endoscopic ultrasound can determine tumor extent and its relationship to adjacent vascular structures. Tumor resectability, however, is limited in many cases, due to invasion of key arteries, such as the pancreatoduodenal artery or SMA, underscoring the importance of adequate preoperative arterial mapping [46]. Although surgery is the treatment of choice in patients with resectable DC, postoperative morbidity remains high, with complications such as infections, pancreatic and biliary fistulas, as well as bleeding from vascular lesions. In terms of adjuvant treatment, chemotherapy with regimens such as capecitabine or folfirinox has been shown to improve survival in patients with complete tumor resection. However, the response to chemotherapy is variable, which highlights the need to consider each case individually. In this context, the identification of anatomical variants in the pancreatic artery acquires a crucial role, since their presence can predispose to iatrogenic lesions in surrounding vascular structures, increasing the risk of surgical complications and affecting postoperative outcomes [47].

As with other tumors of the upper digestive tract, gastric cancer (GC) represents a significant surgical challenge, with arterial irrigation playing a critical role in the planning and execution of procedures. This type of cancer is the fourth leading cause of cancer mortality worldwide and its incidence varies geographically, with the highest rates in East Asia and Central Europe. Despite advances in surgical technology and complementary therapies, the prognosis remains poor in many cases due to late diagnosis. Treatment at specialized centers with expertise in oncological surgery has been shown to improve survival rates, in part thanks to better recognition of vascular anatomical variants that can affect tumor resectability and prevention of intraoperative complications [48,49]. The surgical approach to GC is classified as curative and palliative resection. In curative surgery, the goal is complete removal of the tumor with negative margins, which may require subtotal or total gastrectomy, depending on the extent of the disease. However, safe resection is highly dependent on vascularization of the stomach and its adjacent structures, particularly the CeT and its branches, which include the gastric arteries and the SPDA. Variations in the arrangement of these vessels may influence the feasibility of certain surgical techniques and increase the risk of ischemia in the remaining segments [48,49]. In early stages, endoscopic resection, such as endoscopic submucosal dissection (ESD), offers a minimally invasive alternative for tumors confined to the mucosa or submucosa, without lymphovascular invasion. While these procedures reduce the need for major surgery, they require careful assessment of the risk of vascular involvement in patients with arterial variants, as insufficient perfusion could compromise the integrity of the remaining tissue [50,51]. When the cancer is in advanced stages and complete resection is not possible, palliative procedures aim to relieve symptoms such as obstruction or bleeding. In these cases, techniques such as gastric bypass or stenting can improve the patient’s quality of life. Even in these procedures, however, the arterial anatomy of the pancreas must be considered, as surgical manipulation near the CeT or SMA may predispose to hemorrhagic or ischemic complications [51]. Lymphadenectomy is an essential component in GC surgery, especially D2 lymphadenectomy, which involves the resection of a significant number of lymph nodes and is the standard in Asian countries. However, performing it requires a detailed understanding of pancreatic irrigation, as the proximity of the nodes to the SA and pancreaticoduodenal artery may increase the risk of vascular damage. In this sense, splenectomy has been used in some cases to achieve a more extensive resection, even though recent studies suggest that it does not provide a significant benefit in survival and, instead, increases postoperative morbidity [48]. In patients with locally advanced GC that invades adjacent organs, multivisceral surgery may be necessary to achieve complete resection. Regardless, these interventions have a higher risk of complications, especially when there are anatomical variants in the pancreatic artery. In these cases, preoperative identification of vascular abnormalities is key to reducing the possibility of iatrogenic lesions, which can compromise the perfusion of surrounding structures and increase the risk of complications such as fistulas or postoperative bleeding [52]. Detailed knowledge of pancreatic vascularization is essential not only in oncological surgery, but also in highly complex procedures such as pancreas transplantation. In these cases, surgical planning should consider the anatomical variability of the arteries that supply the pancreas, as these can influence the viability of the graft and the incidence of postoperative complications. This aspect acquires special relevance in patients with type 1 diabetes and comorbidities such as renal failure, in whom pancreatic transplantation represents a key therapeutic option [53]. There are three main pancreas transplant modalities, each with specific implications in terms of arterial irrigation. Simultaneous pancreas-kidney transplantation is the most common procedure in patients with end-stage renal failure, as it allows pancreatic and renal function to be restored in a single procedure. Otherwise, pancreas transplantation after kidney transplantation is performed in patients who have already received a previous kidney graft, while isolated pancreas transplantation is indicated in those with severe metabolic imbalance but with preserved renal function [52]. In all these cases, the arterial supply of the graft must be carefully evaluated, since the presence of anatomical variants can condition the surgical strategy and the patient’s prognosis. The pancreas receives its irrigation through a complex vascular network, derived mainly from the SA, SMA, and GDA. The SA provides pancreatic branches responsible for the perfusion of the body and the tail of the organ, while the pancreatic head depends on a vascular ring formed by the SPDA (branch of the GDA) and the IPDA (branch of the SMA) [53,54]. After all, anatomical studies have shown considerable variability in the pancreatic arterial pattern, which can affect the predictability of vascularization during surgical procedures such as pancreatectomies and transplants. Among the most common variants is the DPA, which can originate from the SA, the CHA, or the SMA. Preoperative identification of these variations is critical to minimize the risk of pancreatic ischemia and reduce the incidence of postoperative complications [54]. Pancreas transplantation is a curative treatment for patients with diabetes or some type of carcinoma. When faced with vascular variants in the living donor or recipient, this must be taken into account for the success of the surgery, since if it is not taken into account, the transplant could fail or the efforts for vascular concordance may be greater. One of the techniques that could help reduce these complications due to vascular variants is normothermic in situ mechanical perfusion. It preserves organs in an optimal physiological state, reduces graft injury, and, given that grafts are metabolically active, offers a platform for graft evaluation and graft repair for suboptimal organs such as those with vascular variants. Therefore, this is considered a technique that can help reduce the probability of vascular complications due to anatomical variants in pancreatic irrigation [55]. Therefore, angiographic or CT angiography (CTA) evaluation plays a key role in surgical planning, allowing surgeons to anticipate potential technical challenges and adopt vascular reconstruction strategies when necessary. In this context, detailed knowledge of arterial irrigation of the pancreas is crucial to optimize outcomes in procedures such as cephalic duodenopancreatectomy (Whipple procedure), segmental pancreatectomies, and pancreatic transplantation. Prior surgical approach decisions based on prior recognition of vascular variants in the pancreatic irrigation will allow the medical team to make informed decisions about the most appropriate approach sites to avoid iatrogenic injuries during surgery. In addition, in the case of more invasive surgeries, decisions must be made about the type of procedure to be performed. Adequate surgical planning based on the patient’s individual vascular anatomy can reduce associated morbidity and improve the safety of these highly demanding procedures [54].

## 4. Discussion

This systematic review with meta-analysis sought to know the anatomical variants in pancreatic irrigation, where we have detailed the anatomy of the different arteries that supply blood to the different regions of the pancreas. The normal anatomy of the irrigation of the pancreas is complex due to its proximity to other surrounding organs of the digestive system that are in contact with the different regions of the pancreas. Furthermore, we have been able to show that if these vessels present anatomical variants, surgical iatrogenic lesions have a greater chance of occurring. Therefore, it is important that surgeons in the abdominal region are aware of these variants, since their prevalence is not low. For this reason, we suggest that they should have anatomical and imaging knowledge of the arteries of the pancreas and know how to identify these variants in the most accurate way in order to provide better treatment to patients. The pancreas is anatomically divided into the head, uncinate process, neck, body and tail. This complex abdominal structure has a complex and organized irrigation, which derives from the CeT and the SMA. The head is supplied by the anterior and posterior pancreaticoduodenal arches, the anterior arch is formed by the ASPDA, a branch of the GDA, and the anterior inferior pancreaticoduodenal artery, a branch of the SMA. The posterior arch follows a similar path, but on the posterior surface of the head. The uncinate process, an extension of the head, is supplied by short vessels that arise directly from the SSA. In the neck, the GDA, which runs to the right, gives rise to the ASPDA. The body is mainly supplied by branches of the SA, which follows a curved path parallel to the upper border of the pancreas, emitting branches such as the DPA and the GPA, which supply the parenchyma. The tail, contained in the splenorenal ligament near the hilum of the spleen, receives its supply from the SA and caudal branches, such as the inferior pancreatic artery [56]. Detailed knowledge of these structures is essential for the discovery of anatomical variants that can pose a challenge when performing surgical procedures and clinical diagnoses. Detailed knowledge of the anatomy of the pancreas and its variants is critical due to its structural complexity and clinical and surgical implications. Variations in irrigation can increase the risk of intraoperative complications, such as bleeding or ischemia, and make surgical procedures such as pancreatoduodenectomy or vascular reconstruction difficult. Planning based on advanced preoperative studies, such as CT or angiography, allows these variants to be identified and thus significantly influence the decisions of the surgical team, improving clinical results and reducing risks associated with interventions. Research in this field plays a crucial role in improving medical and surgical practice by providing a solid foundation for the management of patients undergoing procedures in this anatomical region. Regarding previous investigations carried out, in our search, we have found four reviews that addressed the anatomy of pancreatic irrigation and its anatomical variants. First, we have the review by Bertelli et al. (1998) [15] that analyzed sixteen anatomical studies, of which two coincide with those of our research, with the aim of characterizing the anatomy of the IPDA and contributing original material and angiographic observations. The study highlighted variations in the origin of this artery, which may arise from the SMA, the ARHA, or a common trunk with the first jejunal branches. In clinical terms, the lack of consensus on its origin is highlighted, which complicates surgical procedures such as resection of the head of the pancreas in Whipple surgeries. The authors conclude that understanding this anatomy is crucial to improving surgical outcomes. Our research differs from that conducted by Bertelli et al. (1998) [15] in that the focus of our study encompasses the irrigation of the pancreas in its entirety, while the former study focuses specifically on anatomical analysis and variants in the origin of the IPDA.

Rousek et al. (2022) [7] conducted a meta-analysis of 30 studies, of which 13 coincide with those of our research, to determine the prevalence and origin of the DPA. The main origins were identified as the CeT, the SA, the CHA, and the SMA. From a clinical point of view, the importance of preserving this artery during pancreatic procedures was highlighted to avoid damage to the blood supply to the tail of the pancreas and risks in grafts. The authors conclude that, although the dorsal pancreatic artery is common, its origin and branching are highly variable, highlighting the need for a detailed preoperative study. The research by Rousek et al. (2022) [7] differs from our study, in that their analysis focuses exclusively on the DPA, covering its prevalence, origin, and anatomical variants, while our research comprehensively addresses irrigation of the pancreas [7]. Rebelo et al. (2020) [57] evaluated the outcomes of arterial resection (AR) in pancreatic surgery, including 31 analyses. They found that morbidity and mortality rates in the AR group were 66.8% and 5.3%, respectively. Their study emphasizes that ARs increase the complexity of pancreatic surgery and require multidisciplinary planning. Unlike our study, the main focus of this study is the surgical part, noting that arterial dissections increase morbidity and mortality rates, thus requiring a careful surgical approach. Finally, Xu et al. (2022) [44] reviewed 15 anatomical analyses, of which no studies were found that coincided with those of our research. The authors of this study focused on the hepatic artery and anatomical variants related to pancreatic resection. They found accessory hepatic arteries originating from different points, such as the SMA, the left gastric artery or the abdominal aorta. Clinically, they highlighted that these variants increase the risk of intraoperative lesions, affecting liver perfusion and surgical and oncological outcomes. The authors conclude that accurate recognition of these variants is essential for planning and executing safe and effective surgical procedures. In other circumstances, the study by Xu et al. (2022) [44] differs from ours by focusing comprehensively on accessory hepatic arteries, their incidence, and the impact of these variants on surgical and oncological outcomes after pancreatic resection. While our research also considers the anatomical and surgical implications, it differs by comprehensively addressing pancreatic irrigation, rather than focusing solely on the hepatic arteries and their variants. The results of previous studies show that our review is novel, since we have addressed the variants from a complete and exhaustive anatomical classification. We have also found relevant clinical correlations for surgeries in patients with pancreatic damage or patients who are candidates for transplantation, who will benefit if the vascular flow is known beforehand in diagnostic imaging. This shows that we have detailed several important points for the clinical practice of the retroperitoneal region.

Regarding the results of the meta-analysis and the characteristics of the included studies, the total number of subjects was 1551 for the calculation of prevalence. This n is proportional to the number of studies and is related to the difficulty of imaging the pancreatic vascularization, which is attributed to the limited number of subjects in the primary studies analyzed. Regarding the sample of anatomical variants, most were identified through imaging findings. From a clinical point of view, this highlights the importance of preventing complications in patients who present these variants, thus enabling better intervention or treatment of the region with detailed vascular knowledge. On the other hand, concerning the characteristics of the region where the subjects in the included studies come from, most came from Asia, with more than a thousand subjects from this region. To determine if this variant is mostly related to Asian ethnicity, we analyzed the literature and did not find any study reporting the variant as associated with Asian race or any other. This is an indication that a greater number of subjects may be associated with the greater number of examinations conducted in Asian countries and the large population of this continent. Regarding any association between higher prevalence and sex, it was not possible to verify this, as the n of subjects by sex was similar, and the literature does not report this relationship either. The grouped prevalence of the variant was 11%, while the literature reports variants in pancreatic irrigation at 3 to 7%. Although the data obtained in this review do not fall within the theoretical range, this may be due to the estimates presented by some studies. However, it should be taken into account that the prevalence ranges are close, so for clinicians, variants in pancreatic irrigation should not be considered unusual. Finally, the risk of bias applied to the studies presented a low risk, so the data provided by the studies are reliable and representative for analysis and reporting in this review.

The anatomical variability of pancreatic irrigation has significant clinical implications in surgical planning and in the management of various pathologies, including PC, hepatobiliary interventions, and organ transplantation. A detailed understanding of these variants is critical to minimize intraoperative complications and improve postoperative prognosis. One of the most relevant aspects in pancreatic surgery is the preoperative identification of secondary pancreatic arteries, such as the IPDA and the DPA. IPDA, a branch of the SMA, is key in the irrigation of the pancreas and duodenum. The presence of aneurysms in this artery can generate intestinal angina, manifesting itself with abdominal pain and weight loss, requiring endovascular or surgical treatment to avoid rupture. In addition, DPA contributes to the irrigation of the head and neck of the pancreas, so its identification is crucial in procedures such as duodenocephalopancreatectomy (Whipple procedure), where its preservation is essential to avoid postoperative ischemia. A clinical case has reported the presence of relevant arterial variations, particularly the accessory left hepatic artery originating from the left gastric artery and the emerging left hepatic artery from the GDA. These variants can significantly influence surgical planning, especially in wide resection procedures. A documented clinical case showed retrograde flow to the left liver territory through an accessory artery, which allowed vascular reconstruction to be avoided. Knowledge of pancreatic irrigation is also critical in pancreas transplantation, where the presence of an ARHA may contraindicate simultaneous liver and pancreas transplantation due to difficulty in vascular reconstruction. Further, in patients with exocrine and endocrine pancreatic insufficiency, preservation of minor pancreatic arteries may improve graft perfusion and reduce thrombotic complications. The surgical management of DC and GC is also influenced by the arterial anatomy of the pancreas. Pancreatoduodenectomy is the surgery of choice in tumors of the proximal duodenum, while in distal tumors partial duodenectomy is chosen. Presurgical evaluation of pancreatic irrigation minimizes the risk of ischemia in the remaining segments and reduces complications such as fistulas and hemorrhages.

Since arterial irrigation of the pancreas is highly variable, its clinical relevance is accentuated in surgical procedures such as pancreatoduodenectomy. This procedure, although it is the main strategy in resectable tumors located in the head of the pancreas, is associated with high postoperative morbidity. The presence of arterial anatomical variants can pose a significant challenge for surgeons and affect postoperative outcomes, so identification and preservation of accessory pancreatic and hepatic arteries is critical to reduce risks and avoid ischemic complications in the liver and remaining pancreas. Surgical planning should include a meticulous preoperative study using CTA, MRI, and other imaging techniques. These tools allow vascular variations to be identified and appropriate surgical strategies to be planned, preventing alterations in the distribution of blood flow and reducing the risk of postoperative ischemia. Finally, the preservation of pancreatic blood flow is essential to avoid exocrine and endocrine pancreatic insufficiency. Alteration of arterial supply can lead to endocrine dysfunction, predisposing to secondary diabetes mellitus, while compromise of exocrine function can lead to malabsorption and malnutrition. Therefore, an individualized surgical approach, based on a detailed knowledge of the vascular anatomy of the pancreas, is essential to optimize clinical outcomes and reduce complications in these highly complex procedures [56,57].

Finally, the preservation of pancreatic blood flow is essential to avoid exocrine and endocrine pancreatic insufficiency. Alteration of arterial supply can lead to endocrine dysfunction, predisposing to secondary diabetes mellitus, while compromise of exocrine function can lead to malabsorption and malnutrition. Therefore, an individualized surgical approach, based on a detailed knowledge of the vascular anatomy of the pancreas, is essential to optimize clinical outcomes and reduce complications in these highly complex procedures [56,57,58,59].

### Limitations

This review faced limitations due to potential publication and authorship bias in the included studies. Firstly, studies with differing outcomes that were part of non-indexed literature in the chosen databases might have been overlooked. Secondly, the search methods may have had restricted sensitivity and specificity. Lastly, the article selection was conducted manually by the authors. These factors increase the likelihood of excluding potential cases from regions outside Asia and North America that may not be represented in the scientific literature.

## 5. Conclusions

The discovery of variations in pancreatic irrigation is not an exceptional case, given the large number of blood vessels that contribute to the irrigation of this organ, which is crucial for the correct functioning of several structures of the body system. To perform surgery on the pancreas, it is important to know the different vascular patterns, such as those of the splenic artery and the mesenteric artery, which are key distribution vessels to the pancreas. It must also be taken into account that, in patients undergoing a transplant, a vascular analysis of both the donor and the recipient must be conducted. In the presence of variants in the vascular territory, decisions must be made that can improve blood flow and generate homology in the vascular bed of the recipient. If this cannot be achieved, it may be considered an incompatibility for the transplant. We believe that all these precautions and preventative measures could be improved through the study of more accurate imaging diagnostic tools. Therefore, new studies that improve pre-surgical analysis through imaging will always be necessary.

## Figures and Tables

**Table 1:** Characteristics of studies on arterial variants and their clinical implications in pancreatic and abdominal surgery. Studies included in the systematic review and in the meta-analysis.

Author/Year	G. Region	N and Sample	Age/Sex	Prevalence	Clinical History	Symptoms	Artery with Variants	Description of the Variantas	Clinical Implications
Biehl, 1993 [16]	USA	77 patients	Not reported	9/77 RHA 3/77 HA	Neoplasms, complications of chronic pancreatitis	Not reported	CHA	RRHA or CHA and early bifurcation of the hepatic artery.	Common vascular anomalies may affect surgical strategies, and their appropriate treatment may decrease postoperative morbidity.
Barteli et al., 1998 [15]	Italy	None reported	None reported	Splenic artery (22–80%) Common hepatic artery (12–24%) Celiac trunk (3–33%) Superior mesenteric artery (1.8–25%)	None reported	Not reported	DPA	The DPA has variable origins, commonly arising from the SA, CeT, CHA or SMA.	It can serve as a collateral pathway in cases of celiac-mesenteric arterial stenosis. Additionally, it plays a crucial role in pancreatic surgeries, as it may impact the blood supply to different regions of the organ.
Chio et al., 1993 [17]	USA	1 patient	59/Female	1/1	SMA aneurysm	Epigastric pain and significant weight loss.	IPDA	3 cm IPDA aneurysm	IPDA aneurysm beginning with intestinal angina and weight loss.
Chong et al., 1998 [18]	USA	87 patients	36–75/B	82/87 DPA 9/87 rbDPA 36/87 TPA 45/87 PMA 34/87 CPA 47/87 AA 63/87 PA	Not documented	Not reported	Pancreatic arterial	Visualization of small peripancreatic arteries.	Angiographic depictions of these small vessels were used extensively for the diagnosis and staging of pancreatic ductal adenocarcinoma.
Costea et al., 2019 [19]	Romania	1 patient	61/Male	e1/1	Clinical symptoms of peripheral vascular disease of the lower limb.	Not reported	IPDA	RRHA arising from IPDA, in association with left multiple RAs.	Presence of a RRHA arising from IPDA and right kidney with three RAs [one main RA, one additional RA (AdRA) arising from AA and one accessory RA (AcRA) arising from left common iliac artery].
Di Gregorio et al., 2015 [20]	Italy	1 patient	63/Male	1/1	Hepatocellular carcinoma	Not reported	DPA	RGEA arising from the DPA	According to the study, in abdominal surgery, the identification of the RGEA artery is essential in Whipple duodenocephalopancreatectomy.
Falconer and Griffiths, 1950 [21]	Edinburgh	50 donors	Not specified	IPA: 9/50 SMA 10/50 SPA 1/50 RGA PA: 3/50 CT 6/50 HA 6/50 SA 3/50 SMA	Not documented	Not reported	IPDA and SPDA	Variations in the origin of the IPDA and SPDA.	Not reported
Gordon et al., 1978 [22]	USA	1 patient	49/Male	1/1	Primary amyloidosis	Not reported	DPA	Moderate-sized accessory middle hepatic artery arising from the DPA.	Variations in hepatic blood supply are important to both the surgeon and the radiologist. As these are terminal arteries, surgical ligation of aberrant hepatic arteries can cause liver damage and there is a possibility of gall bladder involvement.
Hong and Freeny, 1999 [23]	USA	27 patients	Not specified	22/27 APDA 22/27 PPDA 26/27 DPA	Not documented	Not reported.	Pancreaticoduodenal arcades and DPA	Variation in the origin of the arteries	None reported
Horiguchi et al., 2008 [24]	Japan	109 patients	62/B	42/105 SA 27/105 CHA 21/105 SMA 9/105 CA 6/105 Others	Pancreatic cancer Biliary tract cancer Intraductal papillary mucinous tumor of the pancreas	Not reported	DPA	Variation in the branching of arteries	Preoperative understanding of the vascular anatomy of the pancreatic head is important in order to reduce intraoperative bleeding.
Huang et al., 2013 [25]	China	63 patients	52/B	15/63 common origin 48/63 noncommon origin	Hepatocellular carcinoma Bleeding Hepatic artery aneurysms	Not reported	RHA	Variation in the origin of the right hepatic artery in relation to the IPDA.	Some centers consider an A/R RHA to be a contraindication to simultaneous liver and pancreas retrieval and transplantation because it represents a challenge to the surgeon who must reconstruct it to avoid dysfunction or graft loss due to hepatic artery thrombosis.
Iede et al., 2018 [26]	Japan	34 patients	65/B	10/34 SMA 8/34 CHA 7/34 SA 1/34 CHA-SMA 1/34 SA-SMA 1/34 CA-SMA	Pancreatic carcinoma, Bile duct carcinoma, Ampullary carcinoma, Neuroendocrine tumor, Serous cystadenoma	None reported	DPA	The right branch or origin of the DPA arising from the CHA, SA, or CA was identified in the first portion of the nerve plexus of the pancreatic head.	According to the study, the findings of the anatomical variations inthe artery allow us to understand why the DPA ligament reduces intraoperative blood loss during PD.
Jiang et al., 2021 [27]	China	13 donors	Females and Males	6/13 SA 5/13 SMA 1/13 CHA 1/13 RGEA	Not reported	Not reported	DPA	The DPA originates, respectively, from the splenic artery, superior mesenteric artery, common hepatic artery and right gastroepiploic artery.	The DPA is one of the major blood supplies to the pancreatic head. A ligation of DPA prior to dissection of the uncinate process can help to completely block the blood supply to the pancreatic head, and therefore improve surgical outcome and safety in LPD.
Kumar et al., 2021 [28]	India	15 donors	18–80/B	ausencia PIPD 1/15 AIPD artery originated from: bifurcation IPDA 11/15; direct branch SMA 2/15; First JA 1/15; second JA 1/15	Chronic pancreatitis, pancreatic cancer, undergoing pancreatic surgery	Not reported	GDA, DPA, and IPDA	GDA originating from right hepatic artery; DPA originating from SA; IPDA branching pattern variation	Variant origin of GDA may alter surgical planning and increase procedural risks, Variant origin of DPA may impact arterial embolization procedures, Knowledge of IPDA variations crucial for accurate surgical approach.
Lin et al., 2012 [29]	China	42 patients	36/B	SA 21/42 SMA 10/42 CHA 5/42	Diabetes received an experimental treatment of autologous bone marrow-derived stem cell transplantation	Not reported	DPA	The DPA may be absent or have rare origins, such as the SMA, rather than the more common sources such as the SA or CA.	Vascular variations can complicate procedures in the pancreas, highlighting the need for precise imaging techniques such as computed tomography angiography to assess vascular anatomy. Knowing these variants is crucial to ensure adequate blood supply to the pancreas and avoid complications during medical interventions.
Macchi et al., 2017 [30]	Italy	10 donors 30 patients	44–81 (4 Males, 6 Females) 70.9 ma (25 Males, 5 Females)	CT: type d 7.7%; type c 3.8% origin DPA: SA 38.5%; CHA 15.4%; CT 7.7%; SMA 3.8% origin TPA; DPA 26.9%; SMA 19.2%; SA 7.7%;PDJ 7.7%; GA 3.8%; GPA 3.8%; PIPD 3.8%	Atherosclerotic pathologies of the abdominal aorta.	Not reported	CeT, DPAand TPA	In 86.7% of cases, the CK was complete, and the most common types were type A (76.9%) and type B (11.5%). In 13.3% of cases, the CK was incomplete presenting a gastrosplenic trunk. The TPA originated mainly from the DPA (26.9%) and the SMA (19.2%), less frequently from other arteries.	Arterial variations can affect the surgical technique used, the management of blood vessels during surgery, and the prevention of postoperative complications, such as pancreatic fistulas.
Marang-van de Mheen et al., 2010 [31]	Netherlands	134 patients	31.7 +/− 12.6 ma	22/134	DM I, insulin dependent	Not reported	DPA	The DPA arises from the CeT or CHA	Not reported
Okahara et al., 2010 [8]	Japan	177 patients	67/B	Major arteries: Replaced RHA 15/162; replaced CHA 3/162; from SMA or CT 1/162; GDA arising LHA 1/162; GEA arising SMA 1/162 Superior ASPDA/PSDA: anastomotic branch ASPDA-DPA 28/129; Double PSPDA from GDA 5/129; PSPDA absent arising replaced RHA 1/129; retropancreatic and/or prepancreatic arcades 26/129. Inferior IPDA/DPA: IPDA from SMA (64%), CT (34%), replaced RHA (1%) or absent (5%); DPA from SMA (1%), AIPDA from SMA (10%) or 1st JA (14%); PIPDA from SMA (12%), 1st JA (9%), DPA (2%) or absent (5%).	Hepatocellular carcinoma, metastatic liver tumor, cholangiocellular carcinoma, other hepatico tumor, bile conduct carcinoma, chronic pancreatitis, and others	Not reported	ASPDA, CHA, and PSPDA	Anastomotic branch between ASPDA and DPA in 14.2% of cases. Presence of a prepancreatic arch causing variations in the opacification pattern on CTA PSPDA originated from the proper hepatic artery.	Implications in the blood supply of the pancreas and in pancreatic interventions. Importance in the interpretation of imaging studies and intervention planning relevant in the treatment of pancreatic cancer and in specific arterial interventions.
Scott et al., 2015 [39]	USA	42 patients	48/B	DPA replaced to SMA 9; DPA arising from DCT 2; DPA arising from CHA 1; CA stenosis 2; CHA replaced to SMA 2; LHA replaced to LGA 7; LHA replaced to CHA 1; RHA replaced to CT 4; RHA replaced to SMA 3.	Occult insulinoma	Hypoglycemic disorders due to endogenous hyperinsulinism are complex to manage. Insulino-ma is the most common cause of hyperinsulinemic hypoglycemia in adults, with an incidence of approximately 4 cases per million per year.	DPA, Common, hepatic, LHA, and RHA	The CT and SMA were abnormal in 38.1% and 35.7% of patients, respectively. Significant variations included DPA replaced by the SMA and celiac stenosis.	Careful review of the pancreatic arterial anat- omy and regional perfusion is critical for correct interpretation of the biochemical results of SACST and improves the sensitivity of localization for occult insulinoma. Importantly, the results of SACST should be interpreted by a multidisciplinary team with expertise in interventional radiology, endocrinology and endocrine surgery.
Shioyama et al., 2001 [32]	Japan	326 patients (304 without pancreatic pathology, 22 pancreatic carcinoma)	54/Male 70/Male	94% PA 72% AA 76% IPDA 96% DPA 41% TPA	Pancreatic carcinoma, duodenal carcinoma, cystic tumor of the pancreas	Not reported	ASPDA and, PSPDA	Variation in the appearance of pancreatic arteries	The difficulty in visualizing arteries such as the GPA, CPA and TPA can complicate the diagnosis and treatment of pancreatic tumors. However, arteries such as the ASPDA and PSPDA, by passing through certain tumors, help to identify duodenal invasion and guide surgical planning in pancreatic carcinoma.
Szuak et al., 2023 [40]	Hungary	50 donors	Not reported	2 PDA 29/50 3 PDA 15/50 1 PDA 1/50 4 PDA 1/50 5 PDA 2/50	The corpses neither had any history of pancreas disease, nor presented any signs of abdominal trauma or macroscopic alteration.	Not reported	Pancreaticduodenals	The SPDA may arise from the left hepatic artery or may have two anomalous branches. There may also be double vascular arches (anterior and posterior) and multiple independent branches from the GDA to supply the head of the pancreas.	Potential risks during surgical procedures due to aberrant origins of the arteries, challenges in surgical planning due to abnormal anatomy, impact on blood flow distribution, and considerations for preserving blood supply during surgical interventions.
Tatsuoka et al., 2021 [34]	Japan	160 patients	70/B	CHA 28/160 SA 27/160 CA 15/160 SMA 27/160 r-RHA 8/160 AIPDA 1/160 MCA 1/160	Pancreatic cancer, bile duct cancer, intraductal papillary mucinous neoplasm	Not reported	DPAramification	Originating from celiac axis or superior mesenteric artery—Branches to uncinate process—Varied ramification patterns; Anomalous origin or branching patterns	In the case of the DPA, early division during Pancreaticoduodenectomy is essential to reduce blood loss by avoiding venous congestion and the potential risk of bleeding complications if branches to the uncinate process are not identified and managed appropriately
Toya et al., 2023 [36]	Japan	148 patients	39 ma ± 10 (62 Males, 66 Females)	FJV dorsal a SMA 128/148 FJV anterior 20/148 IPDA (dorsal) branched (SMA 22/148; FJA 106/148) IPDA(ventral) branched from (SMA 20%; FJA 80%)	pancreatic head cancer	Not reported	IPDA	variants in its origin: distance between the IPDA and cranial mesenteric artery (MCA) based on the anatomy of FJV. In the ventral group, the MCA branched more caudally from the cranial mesenteric artery (SMA), possibly due to anatomical interruption of the MCA branch from the SMA by the ventrally positioned FJV	arterial variations be significant for pancreaticoduodenectomy (PD) surgery and the selection of the AFA (Artery First Approach) technique approach.
Tsutsumi et al., 2013 [37]	Japan	11 donors	Not reported	SA 5/11 SMA 2/11 CHA 1/11 ARHA 2/11 PIPDA 1/11	Not reported	Not reported	DPA	Variable origin: It can arise from the SA, SMA, CHA, accessory right hepatic artery, or PIPDA. Course and distribution patterns may vary; Variable course and distribution pattern.	Understanding arterial variations is essential in surgeries such as pancreaticoduodenectomy to prevent perioperative complications, ensure adequate blood flow and facilitate surgical planning, reducing risks of bleeding and other intraoperative problems.
Yamane et al., 2023 [1]	Japan	110 patients	67/B	type 1 39.43% type 2 15.16% type 3 3.3% type 4 19.21% type 5 8.9% type 6 3.3% type 7 1.1% type 8 1.1% type 9 1.1% type 10 1.1%	Pancreatic disease	Not reported	DPA, AMCA, Arc of Buhler, Arc of Riolan	Variations in the DPA are classified into ten types, depending on their origin and the presence of the AMCA. The main types include DPA originating from the celiac trunk or the SMA, with some cases of special vascular arches (Riolan or Buhler) creating anastomoses with other main arteries.	Arterial variations increase the risk of vascular injury in pancreatic surgeries and may compromise blood flow to specific areas such as the splenic flexure of the colon. It is essential to preserve collateral circulation, such as in the Buhler and Riolan arches, to ensure good flow in cases of mesenteric ischemia or revascularization.
Witte et al., 2001 [38]	Germany	1 donor	89/Female	1/1	None reported	Not reported	DPA additional	The CT gave off four arteries: hepatic, SA, left gastric, and an additional DPA. The DPA joined the SMA forming a longitudinal anastomosis. The APDA andPPDA arches arose from branches of the SPDA and the DPA, whereas the IPDA was absent.	Essential for surgical planning, particularly in procedures involving the pancreas, duodenum, tumor resection, and transplantation. Identifying and assessing the size, location, and course of these variant arteries can help reduce complications.
Woodburne O, 1951 [5]	USA	150 donors	Not reported	Origin ARHB (SMA) 3% Origin MCA (DPA) 5% DPA absent, IPA continuation left branch ASPDA 10% Left branch ASPDA, separate branch SMA 1.3%	Not reported	Not reported	Accessory right hepatic branch	The IPA is generally a branch of the SMA. The arterial arches supplying the pancreas are formed by anastomoses with branches of the SMA system, and their origin may vary between individuals. In addition, the DPA, also known as the MPAin certain contexts, has variations in its nomenclature depending on anatomic descriptions.	Understanding the variability in the origin of the inferior pancreatic artery is essential for surgical planning in pancreatic procedures, as it ensures adequate blood supply to the pancreas. Variations in arterial arches can also affect blood flow, requiring consideration during interventions involving these arterial structures.

**Abbreviations:** AA, anterior arcade; ASPDA, anterior superior pancreaticoduodenal artery; APDA, anterior pancreaticoduodenal artery; CA, celiac artery; CHA, common hepatic artery; CPA, caudal pancreatic artery; CeT, celiac trunk; DPA, dorsal pancreatic artery; GDA, gastroduodenal artery; HA, hepatic artery; IPDA, inferior pancreaticoduodenal artery; PA, posterior arcade; PIPDA, posteroinferior pancreaticoduodenal artery; PSPDA, posterosuperior pancreaticoduodenal artery; PPDA, posterior pancreaticoduodenal artery; PMA, pancreaticomagna; rbDPA, right branch dorsal pancreatic artery; RGEA, right gastroepiploic artery; RHA, right hepatic artery; RRHA, replaced right hepatic artery; SA, splenic artery; SMA, superior mesenteric artery; SPDA, superior pancreaticoduodenal artery; TPA, transverse pancreatic artery.

**Table 2:** Prevalence of variants in the included studies.

Author	Total n	Prevalence
Chong, 1998 [18]	87	9
Horiguchi, 2008 [24]	109	10
Huang, 2013 [25]	63	15
Iede, 2018 [26]	34	3
Jiang, 2021 [27]	13	1
Macchi, 2017 [30]	40	6
Toya, 2023 [36]	148	20
Okahara, 2010 [8]	162	3
Kumar, 2021 [28]	15	3
Szuak, 2023 [40]	50	4
Yamane, 2023 (tipo 2) [1]	110	18
Scott, 2015 [39]	42	9
Woodburne, 1951 [5]	150	6
Shioyama, 2001 [32]	326	2
Lin, 2012 [29]	42	5
Tatsuoka, 2021 [34]	160	28

**Table 3:** Subgroup analysis of the studies included in the meta-analysis.

Parameters	Number Studies and Number of Subjects	Prevalence in (%)	95% CI	I2	*p*-Value
Overall	16 (1551)	11.2	8.18–18.1%	88.12%	-
Cadaveric	5 (238)	14.0	12.12–17.11%	79.11%	0.312
Imaging	12 (1313)	8.84	6.99–10.12%	94.85%	
Asia	11 (1182)	10.8	8.87–11.99%	77.12%	0.0041
África	-	-	-	-	
Europe	2 (90)	1.01	0.77–2.12%	89.77%	
América	3 (279)	2.42	1.41–3.99%	93.12%	
Oceanía	-	-	-	-	
Male	10 (477)	8.31	7.11–9.56%	80.11%	0.12
Female	10 (327)	4.42	2.92–6.11%	78.12%	

## Data Availability

No new data were created or analyzed in this study. Data sharing is not applicable to this article.

## Supplementary Materials

The following supporting information can be downloaded at: https://www.mdpi.com/article/10.3390/medicina61040666/s1, Table S1: Details of the search strategy.
