# Supplementary material for: Anatomical Variants in Pancreatic Irrigation and Their Clinical Considerations for the Pancreatic Approach and Surrounding Structures: A Systematic Review with Meta-Analysis
Source: Medicina (Kaunas). 2025 Apr 4;61(4):666. doi: 10.3390/medicina61040666 (PMC12028877; doi:10.3390/medicina61040666)
Supplement: Supplementary file 1 [file medicina-61-00666-s001.zip › medicina-3511889-supplementary.pdf]

Supplementary Table S1: Details of the search strategy

Database	Search strategy	Results	
		**20-11-24**	**15-02-2025**
Medline	Irrigation of pancreas OR arteries of pancreas AND variations anatomical Filters: English, Humans ((((("irrigant"[All Fields] OR "irrigants"[All Fields] OR "irrigate"[All Fields] OR "irrigated"[All Fields] OR "irrigates"[All Fields] OR "irrigating"[All Fields] OR "irrigational"[All Fields] OR "irrigator"[All Fields] OR "irrigators"[All Fields] OR "therapeutic irrigation"[MeSH Terms] OR ("therapeutic"[All Fields] AND "irrigation"[All Fields]) OR "therapeutic irrigation"[All Fields] OR "irrigation"[All Fields] OR "irrigations"[All Fields]) AND ("pancrea"[All Fields] OR "pancreas"[MeSH Terms] OR "pancreas"[All Fields])) OR (("arterialization"[All Fields] OR "arterializations"[All Fields] OR "arterialize"[All Fields] OR "arterialized"[All Fields] OR "arterializing"[All Fields] OR "arterially"[All Fields] OR "arterials"[All Fields] OR "arterie"[All Fields] OR "arteries"[MeSH Terms] OR "arteries"[All Fields] OR "arterial"[All Fields] OR "arteris"[All Fields] OR "artery"[All Fields] OR "arterious"[All Fields] OR "artery s"[All Fields] OR "arterys"[All Fields]) AND ("pancrea"[All Fields] OR "pancreas"[MeSH Terms] OR "pancreas"[All Fields]))) AND (("variation"[All Fields] OR "variations"[All Fields]) AND ("anatomic"[All Fields] OR "anatomical"[All Fields] OR "anatomically"[All Fields]))) AND ((humans[Filter]) AND (english[Filter]))	94	94
Wos	Irrigation of pancreas OR arteries of pancreas AND variations anatomical Filters: English, Humans	100	101
CINAHL	Irrigation of pancreas OR arteries of pancreas AND variations anatomical Filters: English, Humans	93	95

SCOPUS	Irrigation of pancreas OR arteries of pancreas AND variations anatomical Filters: English, Humans	199	200
Google Scholar	Irrigation of pancreas OR arteries of pancreas AND variations anatomical Filters: English, Humans	50	50
Lilacs	Irrigation of pancreas OR arteries of pancreas AND variations anatomical Filters: English, Humans	37	37
	Total	573	577

* All searches were carried out on February 15, 2025.
